# Time Perspective and Perceived Social Isolation: The Role of Social Interaction Anxiety

**DOI:** 10.3390/healthcare12171736

**Published:** 2024-08-31

**Authors:** Madison E. Stout, Austin R. Medlin, Ritu Gupta, Cindy E. Tsotsoros

**Affiliations:** 1 Department of Psychology, Oklahoma State University, Stillwater, OK 74078, USA; madison.stout@va.gov (M.E.S.); aumedlin@iu.edu (A.R.M.); 2 Center for Health Information and Communication, Health Systems Research, Richard L. Roudebush VA Medical Center, Indianapolis, IN 46202, USA; 3 Department of Health & Wellness Design, Indiana University, Bloomington, IN 47405, USA; 4 HRM & Organizational Behaviour, Indian Institute of Management Raipur, Raipur 492001, India; rgritu@gmail.com; 5 Ryan Research of Neuroscience, Department of Human Development and Family Science, University of Rhode Island, Kingston, RI 02881, USA

**Keywords:** time perspective, social interaction anxiety, perceived social isolation, loneliness

## Abstract

Time perspective is a theoretical construct that describes how humans perceive time, which can influence decision-making and subsequent behavior. Research has shown that an individual’s dominant time perspective can be linked to increased risk of poor health. This study aimed to investigate the relationships between time perspective and perceived social isolation. Specifically, we examined the role of social interaction anxiety in the relationship between time perspective and perceived social isolation in a normative sample of college-aged individuals. Undergraduates (*n* = 1780) at a large midwestern university completed an online survey. Results revealed that future-oriented, past-positive, and present-hedonistic time perspectives were significantly negatively associated with perceived social isolation. In contrast, past-negative was positively associated with perceived social isolation, and these relationships were partially explained by social interaction anxiety. Understanding dominant time perspectives can help us to better assess health risk factors and may help to develop interventions to promote healthy behaviors.

## 1. Introduction

Time perspective (TP) is a theoretical construct that captures an individual’s perceptions of time and describes the extent to which they focus their attention on the past, present, or future, as well as the way their perception may influence their behavior [1]. According to theory, most individuals have a dominant time perspective: past-negative, past-positive, present-fatalistic, present-hedonistic, or future-oriented [1]. TP can be thought of as a personality trait that remains relatively stable over time. However, some research indicates that we can train ourselves to develop a new habitual focus over time (i.e., change our dominant time perspective) [1]. See Gupta and colleagues [2] for more details on each TP domain.

TP can provide an important framework for understanding human motivation and behavior. Future-oriented TP individuals tend to process day-to-day information with planning for the future in mind, overall, reported higher grade point averages, greater life satisfaction and subjective health, lower substance use and risk-taking behaviors, and greater financial knowledge for retirement planning [3]. Additional work suggests that this relationship is mediated by goal monitoring and self-regulation [4]. Present-hedonistic individuals, who process day-to-day information with current pleasure at the forefront of their minds, and present-fatalistic individuals, who process information through a fatalistic lens (e.g., “it’s no use”), have both been associated with higher alcohol, drug, and tobacco use [5], experiencing negative consequences of substance use [6], and higher general risk-taking propensity [7]. Additionally, present-fatalistic individuals tend to have poorer executive control due to increased stress levels and altered emotional states during tasks [8]. Past-negative individuals, who tend to ruminate on negative aspects of the past, tend to report more negative consequences of substance use [6]. In summary, an individual’s dominant TP can have a profound negative or positive effect on their overall physical and mental health, but more research is needed to better understand how TP affects health outcomes. 

One factor associated with poor health that is related to TP and gaining attention is perceived social isolation. Perceived social isolation is the feelings associated with a lack of social connection [9,10], and is closely related to loneliness [11]. Perceived social isolation is thought to be a more chronic or trait-like form of loneliness [12]. Unlike objective social isolation, which is a quantitative measure of social connections, perceived social isolation is determined by examining the quality of an individual’s social and emotional support [9,13]. Relationships between perceived social isolation and negative health outcomes, and even mortality, are especially concerning [10,14,15]. A meta-analysis including 300,000 participants concluded that a lack of social relationships can be as unhealthy as smoking [16]. Although limited, research suggests that TP domains are significantly associated with loneliness across the lifespan [17,18]. Specifically, past-negative, past-positive, and present-hedonistic time dimensions were significantly associated with loneliness in adolescents, such that adolescents with a more negative view of the past and those who tend to focus on positive experiences in the short-term tended to report higher symptoms of loneliness and those with a more positive view of the past tended to report fewer symptoms of loneliness [17]. Recent work during the beginning of the COVID-19 social distancing period in Polish young adults demonstrated similar results (i.e., past-positive was related to lower loneliness, past-negative was related to higher loneliness) and added that future TP was associated with lower loneliness [19]. The current study aims to confirm these associations in a large US college-aged sample. 

A potential mechanism between TP and perceived social isolation is social anxiety. Social anxiety is a persistent and debilitating fear of social situations [20]. The bi-directional relationship between social anxiety and perceived social isolation is well-established across the lifespan [21,22]. Loneliness theory suggests that individuals who perceive themselves to be socially isolated tend to have more negative social cognitions and negative expectations of others, which can then lead to hypervigilance of social threats and, therefore, more negative interactions with others [23]. This theoretical pattern of behavior supports the relationship between social anxiety and loneliness. Few studies to our knowledge have investigated the relationships between TP and social interaction anxiety, however, there is some work associating TP with other types of anxiety and worry. For instance, research has found that future-oriented TP is significantly negatively related to generalized anxiety among adults in urban Turkey [24], Greek males with diagnosed generalized anxiety [25], and Swedish psychiatric outpatients [26]. Additionally, having a dominant past-negative or present-fatalistic TP has been positively linked to both depression and anxiety [27,28], while future-oriented TP has been linked to lower depression and anxiety symptoms [3]. Moreover, people with dominant present-fatalistic perspectives, who tend to believe their life is dictated by fate, are likely to exhibit increased feelings of worry [1]. Conversely, a dominant present-hedonistic perspective, in which individuals are oriented towards enjoyment in the present moment, appears to be a protective factor against depression and anxiety symptoms [28]. An individual’s perception of time can play an important role in their experience of general anxiety and worry, and this study aimed to investigate how TP affects anxiety specifically related to social interactions and feelings of perceived social isolation. 

### Present Study

The current study aims to develop a better understanding of psychological factors and correlates of perceived social isolation. The study was designed to examine the relationships between time perspective, perceived social isolation, and social interaction anxiety. Specifically, we aimed to examine the relationships between TP domains, social interaction anxiety, and perceived social isolation in a large sample of college-aged individuals. We hypothesized that (1) the TP domains past-negative and present-fatalistic would be significantly positively related to perceived social isolation, (2) the TP domains future orientation, past-positive, and present-hedonistic would be significantly negatively associated with perceived social isolation, and (3) social interaction anxiety would partially explain the relationships between all TP domains and perceived social isolation.

## 2. Materials and Methods

### 2.1. Sampling and Procedure

An anonymous questionnaire was administered to 1850 university students at a large Midwestern university. Students enrolled in an introductory psychology course were given the option to participate in the survey, which included measures from multiple other studies. Data collection began and ended prior to March 2020 and was completed through the SONA Systems data management platform, the university’s online research data collection system. Individuals provided informed consent prior to completing the survey. Responses were kept anonymous and only members of the research team had access to the de-identified data. Respondents took an average of 24 min (SD = 10.64) to complete the questionnaire. After submitting their responses, participants received course credit. All procedures followed ethical guidelines and were approved by the university’s IRB.

### 2.2. Scales and Measures

After completing demographic items, participants completed questionnaires measuring the following constructs: (a) the five TP dimensions, (b) perceived social isolation, and (c) social interaction anxiety.

#### 2.2.1. Time Perspective

TP was assessed using the 15-item [2] short form of the Zimbardo Time Perspective Inventory (ZTPI) [1]. This scale is designed to measure the extent to which a person differentially focuses on the past, the present, or the future. Respondents indicate the extent to which each statement accurately describes them using a 5-point Likert-type response format ranging from 1 (strongly disagree) to 5 (strongly agree). This scale is designed to independently measure each of the ZTPI’s five dimensions of TP: past-positive, past-negative, present-hedonistic, present-fatalistic, and future-oriented. Thus, five different subscale scores were derived using this measure. Individuals’ scores for each of the five subscales are calculated as the mean of the three items for each dimension. Higher scores on each subscale indicate greater orientation to time on that dimension. Factor analysis has shown that the scale has five independent factors that correspond to the five hypothesized dimensions [28]. The internal consistencies for the scales used in this study are as follows: past-positive = 0.81, past-negative = 0.69, present-hedonistic = 0.64, present-fatalistic = 0.62, and future-oriented = 0.70. A description of each of the five dimensions can be found in Gupta and colleagues [2].

#### 2.2.2. Perceived Social Isolation

The 6-item Friendship Scale [9] is a self-report measure of perceived social isolation. It has been reported to have sound psychometric properties, including reasonable item-rest-of-test correlations (IRTC) and internal consistency levels (i.e., Cronbach’s alpha). Each item on the Friendship Scale is designed to assess a different dimension of perceived social isolation. All items are answered using a 5-point Likert-type response format ranging from 1 (strongly disagree) to 5 (strongly agree). This scale demonstrated a single-factor structure in the present investigation with a coefficient alpha value of 0.801. Each participant’s perceived social isolation score is the summed score for the six items, with a possible range of 6–30, with higher scores indicating a greater degree of perceived social isolation.

#### 2.2.3. Social Interaction Anxiety

Anxiety related to social interaction was assessed using the 6-item Social Interaction Anxiety Scale−Short Form (SAIS-6; [20]). This short-form scale focuses on the core features of social interaction anxiety, which is considered a more generalized form of social phobia. Responses are scored using a 5-point Likert-type response scale ranging from 0 (not characteristic or true of me) to 4 (extremely characteristic or true of me). The scale has a unitary factor structure and is positively correlated with the original 19-item version of the measure [20]. In the present investigation, the scale showed a single-factor structure with a Cronbach’s alpha of 0.811. The score for each participant is the sum of the six items, with higher scores indicating a greater degree of social interaction anxiety.

#### 2.2.4. Infrequency Scale

**Figure 1:**
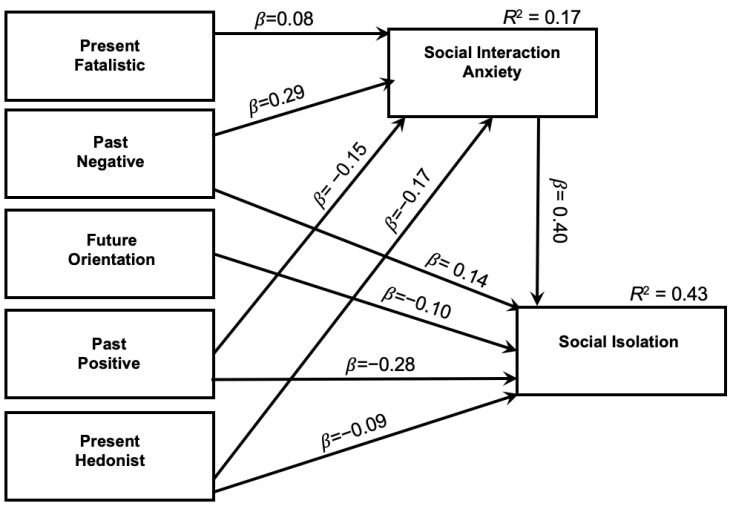
Observed Model for Psychological Predictors of Social Isolation Note. All paths are significant at the 0.01 level.

In addition to the scales and measures from the conceptual model tested in Figure 1, eight additional items were used to measure students’ level of attentiveness while taking the questionnaire. The Infrequency Scale [29] was designed to identify individuals who respond to questionnaire items inattentively by using “catch questions”. All “catch questions” are written in the form of a statement, and the respondents’ task is to indicate the extent to which each statement accurately describes them using a 5-point Likert-type response format from 1 (disagree strongly) to 5 (agree strongly). Individuals who endorsed four or more of the eight items in such a way that suggested inattention were excluded from the investigation.

## 3. Results

### 3.1. Data Cleaning and Descriptive Analyses

Of the original 1850-member sample, 12 individuals were eliminated from the study due to failing four or more attention check questions. Fifty-eight additional individuals (3.1% of the sample) were eliminated from the participant pool for completing the survey in fewer than 12.0 min. Individuals with minimal missing data (i.e., less than 0.05% of the dataset) were retained in the dataset, and in those cases, scale mean scores were imputed as needed for individual items. The final sample size for analysis was 1780.

Frequency distributions and descriptive statistics were computed for each study variable. Most participants were in their first (38.2%) or second (33.9%) year of undergraduate study and identified as female (61.8%). Ages ranged from 18 to 48 years (M = 19.53, SD = 2.38). Most participants self-identified as white (76.2%), with the remaining individuals being African American (7.7%), Native American (5.4%), Hispanic (5.3%), Asian-American (2.0%), or other (3.7%). Table 1 shows mean scores and standard deviations for each construct in the study, as well as their correlations with one another. The data met all assumptions for parametric statistical analysis.

### 3.2. Inferential Analyses

A path analysis model [30,31] was used to test hypotheses for both adjacent predictors and predictors at different levels using AMOS version 25 (IBM, 2018). In addition to reporting *R*^2^ values for each endogenous variable, standardized beta weights (*β*) are reported for each hypothesized path, as well as their corresponding *p*-values. The observed path model is shown in Figure 1. All statistically significant effects are reported in the text below, irrespective of their magnitude.

### 3.3. Path Analysis Model

This path analysis was used to examine possible mechanisms of the association between TP domains and perceived social isolation, and more specifically, the viability of the hypotheses set in the introduction. Recommendations by Brown [32] were used to evaluate goodness-of-fit for each model. CFI, TLI, and AGI values were adequate (i.e., all greater than 0.90), the chi-square to degrees of freedom ratio was less than 2.0, and RMSEA (error) values were between 0.08 and 0.10.

The first development of the path model was fully saturated. As expected, this model was a somewhat poor fit to the data, and some fit indices could not be computed: χ^2^(0) = 0.00, χ^2^/DF = 0.00, CFI = 1.00, GFI = 1.00, and RMSEA = 0.215 (0.206; 0.223). The fully saturated model indicated that certain paths should be removed. After removing two of the paths, the present model revealed an acceptable set of fit indices: χ^2^(4) = 2.03, *p* = 0.73, χ^2^/DF = 0.51, CFI = 1.00, GFI = 1.00, TLI = 1.000, and RMSEA = 0.000 (0.000; 0.026). The observed effects were reasonable to expectations, with most of the TP domains being related to social interaction anxiety and perceived social isolation.

The five TP domains explained forty-three percent of the overall variance in perceived social isolation. At the same time, after accounting for perceived social isolation, the five TP domains accounted for 17% of social interaction anxiety (see Figure 1). Social interaction anxiety was significantly positively related to perceived social isolation (ß = 0.40). As hypothesized, this suggests that individuals who report higher symptoms of social interaction anxiety also report feeling more socially isolated.

Present-fatalistic TP was significantly positively related to social interaction anxiety (ß = 0.08) but was not related to perceived social isolation after accounting for social interaction anxiety. This indicates that individuals who tend to think “It’s no use”, may be more likely to report anxiety around social interactions. Future-oriented TP was significantly negatively associated with perceived social isolation (ß = −0.10) and was not significantly related to social interaction anxiety, suggesting that individuals who, unlike present-fatalistic thinkers, tend to plan for their future may be less likely to report being socially isolated.

Past-negative TP was significantly positively related to social interaction anxiety (ß = 0.29) and perceived social isolation (ß = 0.14). This suggests that individuals who tend to focus more on negative aspects of the past were also more likely to report social interaction anxiety and, in turn, perceived social isolation. Conversely, past-positive TP was negatively related to social interaction anxiety (ß = −0.15) and perceived social isolation (ß = −0.28), suggesting that individuals who tend to think fondly of their past may be less likely to report social interaction anxiety and, in turn, may feel less socially isolated. Finally, present-hedonistic TP was negatively associated with social interaction anxiety (ß = −0.17) and perceived social isolation (ß = −0.09), suggesting that individuals who tend to focus on enjoying the present moment may be less likely to report anxiety with social interactions and, in turn, less perceived social isolation.

## 4. Discussion

The current study examined the relationships between TP domains, social interaction anxiety, and perceived social isolation in a large sample of college-aged individuals. The overarching goal of this study was to examine how the five TP domains relate to perceived social isolation, and whether social interaction anxiety may play a role in these relationships. It was hypothesized that past-negative TP and present-fatalistic TP would be significantly positively related to perceived social isolation; future-oriented TP, past-positive TP, and present-hedonistic TP would be significantly negatively associated with perceived social isolation; and social interaction anxiety would partially explain the relationships between all TP domains and perceived social isolation.

### 4.1. Time Perspectives and Perceived Social Isolation

Results of the present study revealed that four TP domains were related to perceived social isolation. As hypothesized, future-oriented, past-positive, and present-hedonistic TPs were associated with lower perceived social isolation scores. These findings suggest that an individual is less likely to report feelings of loneliness if they tend to (a) plan ahead and think about the future, (b) look on their pasts fondly, and/or (c) focus more on enjoying the present. Results are largely consistent with existing literature, which has found links between future-oriented TP and reduced loneliness [19,33]. Studies suggest that feeling one’s future is limited (i.e., low future-oriented TP) may decrease efforts to establish or maintain quality relationships [33], and thus increase perceived social isolation.

Additional research is needed to support the associations between perceived social isolation and past-positive and present-hedonistic TPs. However, Loneliness Theory [23] provides important insight into how past-positive and present-hedonistic TPs may be associated with lower perceived social isolation in the present study. This theory suggests that lonelier individuals tend to have more negative expectations about social interactions and, therefore, tend to be more hypervigilant to social rejection and other social threats. Individuals who are hypervigilant to social threats may be more likely to perceive an insult or rejection from a neutral statement. This means that they may be more likely to leave a social interaction feeling slighted, which can increase loneliness and inform the expectations they bring to their next social interaction [23]. Individuals with past-positive and present-hedonistic TPs do not tend to ruminate on negative experiences from the past [1]. These individuals may tend to feel less lonely because (1) they are less focused on negative experiences from the past, (2) they may tend to bring more positive or neutral expectations into social interactions, or (3) they are less hypervigilant to social threats. Past-positive and present hedonistic TPs may not only be related to lower current feelings of perceived social isolation but may put individuals at lower risk of becoming lonely.

Conversely, in the present study, past-negative TP was positively related to perceived social isolation, in line with hypotheses. This finding is consistent with another study demonstrating that past-negative TP is linked to increased loneliness during social distancing due to the COVID-19 pandemic [19]. As described above, loneliness theory [23] may provide some insight into how negative perceptions of the past be related to increased loneliness. It was hypothesized that present-fatalistic TP would also be positively associated with perceived social isolation, but results suggested there was not a significant relationship. Given that perceived social isolation is defined as the deficit between one’s desire for social connection and one’s actual feelings of social connection, it makes sense that individuals who believe they cannot influence the future may tend to have differing perceptions of their social relationships. This lack of association is somewhat supported by previous work. Although perceived social isolation was not examined, in their 2013 study, Bernstein and Benfield found no associations between present-fatalistic TP and sensitivity to rejection [34], which could be considered a close correlate of loneliness. At the same time, although findings do not suggest present-fatalistic TP is not linked to feelings of loneliness, it may still act as a notable risk factor. In fact, research does link present-fatalistic TP with higher perceived stress [25]. More research is needed to understand the potential health effects of present-fatalistic TP.

### 4.2. The Role of Social Interaction Anxiety

Social interaction anxiety was investigated as a factor that may explain the cross-sectional relationship between time perspective and perceived social isolation. As hypothesized, social interaction anxiety was associated with greater feelings of perceived social isolation, independent of TP. The bi-directional relationship between social interaction anxiety and perceived social isolation is well established in the literature [21,22]. This finding is consistent with the theoretical understanding of the relationship between social interaction anxiety and perceived social isolation [23,35] as well as with previous studies, which have reported that individuals who report feeling socially anxious also report higher perceived social isolation than those who are not socially anxious [36].

This study provides novel evidence to support the role of social interaction anxiety in the relationship between some TP domains and perceived social isolation. Specifically, the past-positive and present-hedonistic domains were related to lower social interaction anxiety scores, suggesting that individuals who are more nostalgic or are more oriented towards experiencing pleasure in the present tend to experience lower social interaction anxiety and, in turn, lower perceived social isolation. Further, present-fatalistic and past-negative domains were related to higher social interaction anxiety scores, suggesting that individuals who tend to believe the future to be determined by fate and those who have more aversive feelings about their past will experience more social interaction anxiety, and in turn greater perceived social isolation. However, future-oriented TP was not associated with social interaction anxiety, which is not consistent with the literature on generalized anxiety and worry [24,25]. As noted, there were no previous studies investigating dominant TP and social anxiety. Previous work has examined worry and generalized anxiety, which may explain any differences in findings. Overall, these findings suggest that future work should longitudinally investigate social interaction anxiety as a mediator between dominant TP and perceived social isolation.

### 4.3. Practical Implications: Time Perspective Therapy

These findings are notable and suggest that the way individuals perceive time may be a risk factor or protective factor for perceived social isolation, and that these relationships may be partially explained by social interaction anxiety. As demonstrated by the literature, being at high risk for perceived social isolation can increase your risk of all-cause mortality [15,37], and thus, treatment for perceived social isolation may reduce health risk. Although research is limited, the most-supported treatment option at this time for reducing symptoms of perceived social isolation and social interaction anxiety is cognitive behavioral therapy (CBT). CBT is one of the most evidence-based and most commonly used psychotherapies and is used to reduce symptoms of social anxiety and other psychological disorders. Research suggests that CBT-based interventions are the most effective treatments for reducing loneliness [38]. As such, CBT is a strongly supported option for reducing social interaction and anxiety and perceived social isolation. However, high dropout rates for CBT are noteworthy [39,40], and suggest that many patients may discontinue treatment before experiencing maximal benefit. For patients who dislike CBT, having another empirically supported treatment option is necessary.

This study provides preliminary support for future investigations of another method for reducing perceived social isolation: time perspective therapy (TPT) [41]. TPT is a version of narrative therapy and was developed to treat post-traumatic stress disorder by helping patients to establish a balance between all TPs [41]. A recent study among Iranian women with breast cancer suggested that, compared to the control group, TPT significantly reduced post-traumatic stress symptoms, anxiety, and depressive symptoms [42]. TPT has also been shown to reduce depressive symptoms in adolescents [43] and suicidal ideation in adolescents [44]. To our knowledge, there is no existing research examining the effectiveness of TPT to reduce perceived social isolation or social interaction anxiety. However, a meta-analysis revealed that the most effective treatments for perceived social isolation address maladaptive thoughts and perceptions about social interactions [38]. Similar to CBT, an aim of narrative therapy is to address maladaptive “self-narratives”. As such, from a theoretical perspective, TPT may be effective in reducing perceived social isolation. More research is needed to support this hypothesis.

### 4.4. Limitations

This study should be interpreted considering its limitations. First, our sample comprised a college-aged sample of students in a Midwestern University in the United States. Future studies should investigate these relationships in a more representative sample to promote generalizability. Next, although associations were significant at the 0.01, no additional statistical analyses were performed to correct for common method bias. Additionally, Cronbach’s alphas for three TP domains were below the recommended cut-off of 0.07, which could represent random responding from participants or poor scale design. Lastly, the variables in the present study were measured cross-sectionally. The findings from this preliminary study provide promising theoretical support for future, longitudinal investigations of the relationships between TPs, social anxiety, and perceived social isolation, as well as future intervention studies measuring the efficacy of TPT in treating perceived social isolation and social interaction anxiety. Future studies should also investigate important health outcomes (e.g., cardiovascular diagnoses), as well as important health behaviors (e.g., healthy eating, exercise, smoking).

## 5. Conclusions

The present study investigated the relationship between time perspective (TP) domains and perceived social isolation and explored the role of social interaction anxiety. Analyses revealed that past-negative TP was related to higher perceived social isolation, while future-oriented, past-positive, and present-hedonistic were related to decreased perceived social isolation. Additionally, present-fatalistic, past-negative, and present-hedonistic TPs were positively associated with social interaction anxiety, while past-positive was associated with lower social interaction anxiety. Future studies should investigate social anxiety as a mediator between TP and perceived social isolation. Findings suggest that the Zimbardo Time Perspective Inventory (ZTPI) could help to understand an individual’s risk for perceived social isolation and, therefore, their risk for associated negative health outcomes. At the same time, this study provides preliminary evidence that time perspective therapy should be explored as a potential treatment for perceived social isolation or social anxiety.

## Figures and Tables

**Table 1:** Pearson Correlation Matrix, Means, and Standard Deviations.

	1	2	3	4	5	6	7
1. Perceived social isolation	--						
2. Social interaction anxiety	0.54 **	--					
3. Past-positive	−0.47 **	−0.27 **	--				
4. Past-negative	0.36 **	0.34 **	−0.31 **	--			
5. Future-oriented	−0.18 **	−0.06 *	0.19 **	−0.06 *	--		
6. Present-fatalistic	0.08 **	0.11 *	−0.03	0.13 **	−0.07 **	--	
7. Present-hedonistic	−0.20 **	−0.17 **	0.18 **	0.06 **	0.00	0.12 **	--
Mean:	13.16	4.12	3.50	2.99	3.45	2.45	3.16
Standard Deviation:	4.57	3.98	0.94	0.96	0.87	0.84	0.83

Note. * *p* < 0.05, ** *p* < 0.01.

## Data Availability

Data are unavailable due to privacy reasons.
